# The Impact of Maternal Dietary Avoidance During Breastfeeding on Physical Growth and Social–Emotional Development in Infants with Food Allergies: A Prospective Cohort Study

**DOI:** 10.3390/children13050603

**Published:** 2026-04-27

**Authors:** Jun Fang, Rui’an Wang, Zhanzhan Zhang, Yuanfeng Zhong, Yannan Wan, Qian Chen, Xuelin Xia, Xuan Zhang

**Affiliations:** Department of Growth, Development and Mental Health of Children and Adolescence Center of Children’s Hospital of Chongqing Medical University, National Clinical Research Center for Children and Adolescents’ Health and Diseases, Ministry of Education Key Laboratory of Child Development and Disorders, Chongqing Key Laboratory of Child Neurodevelopment and Cognitive Disorders, Chongqing 400014, China; fangjun@stu.cqmu.edu.cn (J.F.); ruian6346@gmail.com (R.W.); zhangzhanzhan@hospital.cqmu.edu.cn (Z.Z.); zhongyf@hospital.cqmu.edu.cn (Y.Z.); wanyannan@stu.cqmu.edu.cn (Y.W.); chenqian.1986@hospital.cqmu.edu.cn (Q.C.); xuelinxia@hospital.cqmu.edu.cn (X.X.)

**Keywords:** food allergy, breastfeeding, dietary avoidance, infant growth, social–emotional development

## Abstract

**Highlights:**

**What are the main findings?**

- Extensive maternal dietary avoidance (≥5 food categories) during breastfeeding of infants with food allergy is associated with suboptimal linear growth at 12 months.
- Higher maternal dietary avoidance is linked to increased social–emotional risk scores (ASQ:SE-2) in infants.

**What are the implications of the main findings?**

- Infants whose mothers practice extensive dietary avoidance should be recognized as a high-risk group requiring integrated nutritional and developmental monitoring.
- Clinical management of food allergy in breastfed infants should balance allergen avoidance with adequate maternal nutrition and routine developmental surveillance.

**Abstract:**

**Background:** Maternal dietary avoidance during breastfeeding of infants with food allergies (FA) is common, but its impact on growth and development is unclear. **Methods:** This prospective cohort study enrolled infants aged 4–6 months who were mainly breastfed from the child health care clinic of Children’s Hospital of Chongqing Medical University, Chongqing, China. The participants were classified into a group with clinically diagnosed FA and a non-food allergies (NFA) group. To analyze avoidance extent, the FA group was stratified into high (≥5 types) and low (<5 types) avoidance subgroups based on the median number of avoided food categories. Outcomes included anthropometric Z-scores and ASQ:SE-2 social–emotional scores at 12 months, analyzed using Generalized Estimating Equations. **Results:** High avoidance mothers comprised 50% of the FA group. Compared to the high avoidance group, the non-FA group showed significantly better linear growth (β = 0.84, 95% CI 0.38–1.30, *p* < 0.001). Weight and head circumference showed no differences. High avoidance was associated with higher ASQ:SE-2 scores versus both the low avoidance and non-FA groups (*p* < 0.001), indicating greater social–emotional concerns. **Conclusions:** Extensive maternal dietary avoidance was associated with suboptimal linear growth and higher social–emotional risk scores in infants with FA, underscoring the need for integrated nutritional and developmental monitoring. These findings warrant cautious interpretation given the exploratory nature of this single-center study.

## 1. Introduction

Food allergy in children has become a significant global public health concern, with prevalence rates rising steadily over the past two decades [1]. In infants and young children, the most commonly implicated allergens include cow’s milk, hen’s egg, peanut, tree nuts, soy, wheat, fish, and crustacean shellfish [2]. For predominantly breastfed infants, allergens ingested by the mother can be secreted into breast milk and may trigger allergic symptoms in sensitized individuals [3,4]. This intersection between breastfeeding and maternal dietary management represents a critical clinical challenge in the care of food-allergic infants.

Current international guidelines consistently recommend that breastfeeding should not be routinely discontinued in infants with FA, as breast milk provides essential nutritional and immunological benefits [2,5]. Chinese evidence-based guidelines similarly emphasize the importance of continued breastfeeding in this population [6]. Despite these recommendations, many lactating mothers implement dietary eliminations. These eliminations are often extensive and self-directed, driven by concerns about their infant’s tolerance or by medical advice [7]. In clinical practice, the most frequently avoided foods are cow’s milk, hen’s egg, and shellfish, though avoidance of multiple food categories is common. This discordance between guideline recommendations and real-world maternal dietary behavior highlights the need to evaluate the potential consequences of such elimination diets.

Emerging evidence suggests that extensive maternal dietary avoidance may have unintended effects on both maternal and infant health. Maternal nutritional intake during lactation influences the composition of breast milk, particularly essential fatty acids, calcium, vitamin D, and other micronutrients [8,9]. Restrictive maternal diets, including vegan or extensive multi-food elimination diets, can alter breast milk nutrient profiles [10,11]. These alterations may subsequently affect infant outcomes, including gut microbiota development and growth [12,13]. However, the existing literature has focused primarily on breast milk composition itself or on short-term allergic symptom resolution. Prospective data examining the effects of maternal dietary avoidance on longitudinal physical growth and social–emotional development in infants with FA remain limited.

Furthermore, it is unclear whether the extent of maternal avoidance, rather than simply its presence or absence, shows a graded association with infant developmental outcomes. To our knowledge, this is the first prospective study to simultaneously examine both linear growth trajectories and social–emotional development, assessed via the ASQ:SE-2, in breastfed infants with FA, stratified by the breadth of maternal dietary elimination. The novel aspects of this study include: (1) the use of a validated social–emotional screening tool in this specific clinical population; (2) stratification by the number of avoided food categories rather than binary avoidance status; and (3) simultaneous evaluation of physical growth and developmental outcomes in a single cohort.

The present prospective cohort study was designed to evaluate the association between the extent of maternal dietary avoidance and two critical domains of infant health. The first domain is physical growth, assessed via World Health Organization anthropometric Z-scores. The second domain is social–emotional development, assessed via the Ages and Stages Questionnaires Social Emotional, Second Edition (ASQ:SE-2). This study specifically examined infants with clinically diagnosed FA whose mothers practiced varying degrees of dietary elimination, compared with a non-food-allergic control group. It was hypothesized that a greater number of avoided food categories would be associated with suboptimal linear growth and higher social–emotional risk scores at 12 months of age. By stratifying infants according to the breadth of maternal dietary elimination, this study aimed to identify a high-risk group requiring integrated nutritional and developmental surveillance.

## 2. Methods

### 2.1. Study Design and Participants

This prospective cohort study recruited infants aged 4–6 months from the Child Health Care Department of Children’s Hospital of Chongqing Medical University, Chongqing, China, between October 2023 and February 2025. The participants were identified through two principal strategies: referral by attending pediatricians during routine well-child visits and self-referral by parents in response to study posters displayed in the clinic waiting area. All potentially eligible mother-infant pairs were assessed against the predefined inclusion and exclusion criteria. Eligible participants were mother–infant pairs who fulfilled the predefined criteria, with written informed consent obtained.

Inclusion criteria: (1) Predominantly breastfed infants aged 4–6 months at enrollment; “predominant breastfeeding” was defined as breast milk being the primary source of nutrition, including infants who were exclusively breastfed or for whom breast milk remained the dominant component of their diet despite the introduction of limited complementary foods (e.g., infant cereal, purees); (2) full term, with birth weight between the 10th and 90th percentiles; (3) no major congenital or chronic diseases; (4) long-term residents of Chongqing, China.

Exclusion criteria: (1) Hospitalization > 30 days during the neonatal period; (2) development of major/chronic illness or surgery during follow-up; (3) caregiver with diagnosed psychological/psychiatric problems; (4) inability to complete follow-up procedures; (5) development of any allergic disease in the control group during the follow-up period, which would necessitate reclassification of the infant’s allergy status.

Infants were classified into two groups. The FA group was identified through standardized clinical assessment by senior pediatricians, requiring consistent parental report of clinically significant symptoms temporally related to food ingestion and marked improvement after maternal elimination of the suspected food. This clinical diagnostic approach was consistent with the Chinese evidence-based guidelines for food allergy in children [14]. According to these guidelines, a diagnosis of FA may be established based on a clear and reproducible history of symptom onset following food ingestion and symptom resolution upon dietary elimination, particularly when an oral food challenge is not feasible or clinically indicated. This pragmatic diagnostic strategy reflects real-world clinical practice in pediatric allergy care. The NFA group consisted of infants with no history of allergic diseases or food-related adverse reactions. Mothers in this group did not practice dietary avoidance related to infant symptoms.

To analyze avoidance extent, the FA group was stratified into high (≥5 types) and low (<5 types) avoidance subgroups based on the median number of avoided food categories. This cutoff was selected as an exploratory threshold given the absence of an established clinical definition for “extensive” dietary avoidance. The median split allowed balanced subgroup sizes for preliminary comparisons and was not intended to represent a clinically validated cutoff.

### 2.2. Data Collection

All questionnaires were administered by trained research staff through face-to-face interviews with mothers during routine clinic visits. Interviews were conducted in a private consultation room within the Child Health Care Department to ensure confidentiality and consistency. Anthropometric measurements were performed by trained nurses at each visit. The infants were followed up monthly from enrollment (4–6 months of age) until 6 months of age, and every two months from 7 to 12 months of age. At each follow-up visit, anthropometric measurements were obtained and questionnaires were administered.

#### 2.2.1. Basic and Clinical Information

A structured questionnaire was used to collect demographic and perinatal data, including infant sex, birth anthropometrics, gestational age, delivery mode, parental age, education level, and family allergy history.

#### 2.2.2. Physical Measurements

Trained nurses measured infant weight, length, and head circumference at each visit. Z-scores were derived using WHO Anthro software (version 3.2.2) based on WHO Child Growth Standards.

#### 2.2.3. Assessment of Maternal Diet

Feeding practices (breastfeeding, complementary food introduction, supplement use) and maternal dietary avoidance were assessed via a questionnaire. Mothers reported complete elimination (≥2 weeks) of eight common allergenic food categories (cow’s milk/dairy, hen’s egg, peanuts, tree nuts, wheat, soy, fish, crustacean shellfish). Infant dietary avoidance was also recorded. Mothers in the FA group reported dietary avoidance either on the advice of a physician or on their own initiative due to suspected or confirmed infant food allergy. Mothers in the NFA group did not practice any dietary avoidance.

#### 2.2.4. Assessment of FA Symptoms

A symptom questionnaire based on Chinese expert consensus [14] was used to record allergy-related symptoms (skin, gastrointestinal, respiratory, behavioral) temporally linked to food ingestion. Family allergy history was collected.

#### 2.2.5. Assessment of Infant Social–Emotional Development

The Ages & Stages Questionnaires: Social-Emotional, Second Edition (ASQ:SE-2) was used at each follow-up [15]. This parent-completed tool screens social–emotional competencies across seven behavioral domains. Higher total scores indicate greater developmental concern. Detailed administration procedures, item structure, and cutoff scores are provided in Supplementary Method S1. It should be noted that the ASQ:SE-2 is a screening tool designed to identify children at risk for social–emotional difficulties. Elevated scores indicate the need for further assessment and do not constitute a clinical diagnosis.

### 2.3. Ethics

This study was approved by the Ethics Committee of Children’s Hospital of Chongqing Medical University (Approval No.: (2023) Ethics Review (Research) No. (366)). It was registered in the Chinese Clinical Trial Registry (Registration No.: ChiCTR2500111228). Written informed consent was obtained from all guardians.

### 2.4. Statistical Analysis

Data were analyzed using SPSS 29.0. The normality of continuous variables was assessed using Shapiro–Wilk tests and visual inspection of Q-Q plots. For variables deviating from normality, non-parametric tests (Mann–Whitney U test) were applied. Continuous variables are presented as mean ± SD or median (IQR). Categorical variables are presented as frequency (%). Group comparisons used *t*-tests, Mann–Whitney U tests, or chi-square tests. Longitudinal analyses used Generalized Estimating Equations (GEE) with an exchangeable correlation structure. The primary models examined the association between group status (FA high-avoidance, FA low-avoidance, and NFA) and outcomes, including length-for-age Z-score and ASQ:SE-2 total score. Models were adjusted for infant age (continuous), infant sex, birth weight Z-score, and mid-parental height. These covariates were selected a priori based on their established biological relevance to infant growth and development. Due to the modest sample size, additional covariates such as maternal nutritional status, complementary feeding timing, and socioeconomic factors could not be simultaneously included to avoid model overfitting. The potential for residual confounding from these unmeasured factors is acknowledged in the Discussion.

Linear regression assessed factors influencing maternal avoidance. Statistical significance was set at α = 0.05 (two-sided). Given the multiple comparisons conducted across different growth parameters and time points, we acknowledge an increased risk of Type I error. We therefore focused interpretation on effect sizes and confidence intervals rather than statistical significance alone.

#### Sample Size Calculation

Sample size was calculated using PASS software (version 2021) based on the primary outcome of length-for-age Z-score at 12 months. The required sample size was 145 mother-infant pairs per group after adjusting for 15% loss to follow-up. Due to single-center constraints, final enrollment (40 FA, 35 NFA) was below this target, making this an exploratory study (see Supplementary Method S2 for detailed calculations).

## 3. Results

### 3.1. Study Cohort Characteristics and Follow-Up Compliance

The study cohort comprised 75 mother–infant pairs, stratified into a FA group (*n* = 40) and a NFA group (*n* = 35). As presented in Table 1, baseline demographic, perinatal, and parental characteristics were comparable between groups (all *p* > 0.05). The participant flow is summarized in Supplementary Figure S1.

### 3.2. Patterns and Determinants of Maternal Dietary Avoidance

All 40 mothers in the FA group reported some degree of dietary avoidance, either on the advice of a doctor or on their own initiative, due to their infant’s food allergy status. Mothers in the NFA group did not practice any dietary avoidance. The most commonly avoided foods were cow’s milk/dairy products (85.0%) and crustacean shellfish (80.0%) (Table 2, Part A). Half of the mothers (50.0%) avoided five or more food categories (Table 2, Part B). Linear regression analysis indicated that the number of infant allergic symptom systems was the only factor significantly associated with a greater number of food avoidances (β = 0.64 per additional system, *p* = 0.03) (Table 3).

### 3.3. Association Between Maternal Dietary Avoidance and Infant Physical Growth

**Figure 1:**
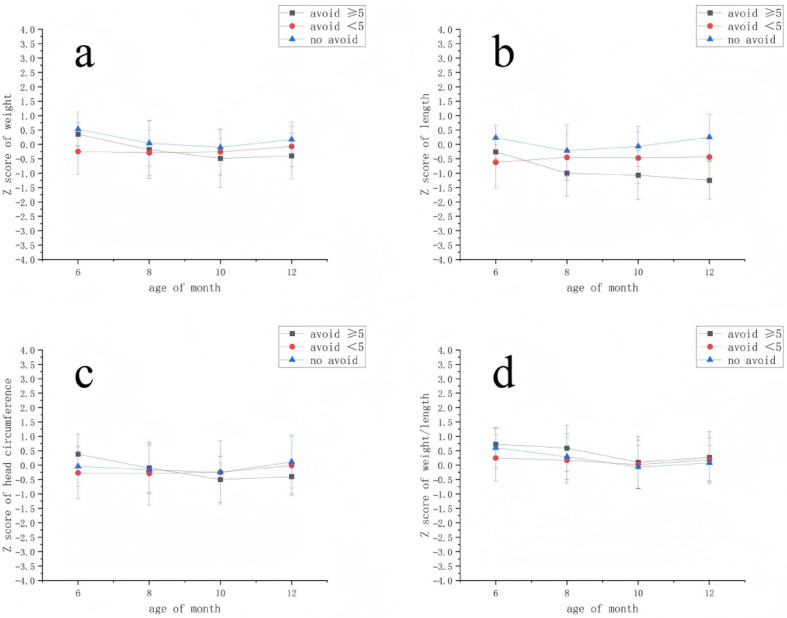
Longitudinal trajectories of growth Z-scores by maternal dietary avoidance group. (**a**) Weight-for-age Z-scores. (**b**) Length-for-age Z-scores. (**c**) Head circumference-for-age Z-scores. (**d**) Weight-for-length Z-scores. Data points represent the mean Z-score, and error bars represent the standard deviation. The sample size (n) for each group at each time point is provided in Supplementary Table S1. Group definitions: FA High-Avoidance (black): infants with food allergy whose mothers avoided ≥5 food categories; FA Low-Avoidance (red): infants with food allergy whose mothers avoided <5 food categories; NFA (blue): infants with no food allergy. Mothers in this group did not practice dietary avoidance.

Longitudinal analysis using Generalized Estimating Equations (GEEs) revealed a significant association between maternal avoidance and infant linear growth (Figure 1). After adjustment for infant age, the NFA group showed higher length-for-age Z-scores compared to the FA high-avoidance subgroup (length-for-age Z-score difference: β = 0.84, 95% CI 0.38 to 1.30, *p* < 0.001) (Table 4). The difference between the FA low-avoidance and high-avoidance subgroups did not reach statistical significance (β = 0.50, 95% CI −0.09 to 1.08, *p* = 0.10). Associations for infant weight, head circumference, and weight-for-length Z-scores were not statistically significant in the models (all *p* > 0.05). Detailed longitudinal anthropometric data are provided in Supplementary Table S1.

### 3.4. Association Between Maternal Dietary Avoidance and Infant Social–Emotional Development

**Figure 2:**
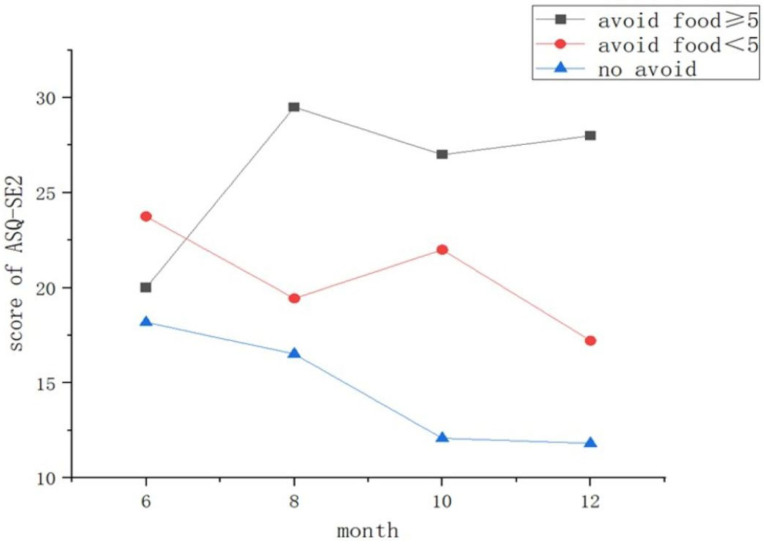
Developmental trajectories of social–emotional scores by maternal dietary avoidance group. The plot shows the median Ages & Stages Questionnaire: Social-Emotional, Second Edition (ASQ:SE-2) total score at each follow-up visit. A higher score indicates greater concern regarding social–emotional development. Group definitions: FA High-Avoidance (black): infants with food allergies whose mothers avoided ≥5 food categories; FA Low-Avoidance (red): infants with food allergies whose mothers avoided <5 food categories; NFA (blue): infants with no food allergies. Mothers in the NFA group did not practice dietary avoidance.

Maternal dietary avoidance was also significantly associated with infant social–emotional development (Figure 2). Compared to the FA high-avoidance group, infants in the NFA group had significantly lower (more favorable) ASQ:SE-2 scores (β = −13.34, 95% CI −18.39 to −8.30, *p* < 0.001). Infants in the FA low-avoidance group also showed significantly better scores than those in the high-avoidance group (β = −7.66, 95% CI −14.07 to −1.25, *p* = 0.02) (Table 4). A detailed distribution of ASQ:SE-2 scores at each time point is provided in Supplemental Table S2.

## 4. Discussion

Many mothers of breastfed children with FA choose to avoid certain foods or use special formula. However, research and guidelines suggest that it is not recommended to stop breastfeeding easily for infants with FA [16]. Therefore, it is important for mothers to carefully monitor their diet and regularly assess their infant’s growth and nutrition to ensure proper development. This study examined the impact of maternal dietary avoidance on the physical growth and social–emotional development of infants. The results indicated that maternal food avoidance was associated with suboptimal linear growth and higher social–emotional risk scores. Given the observational nature of this study, the reported associations should not be interpreted as causal. Maternal dietary avoidance likely serves as a marker of greater initial allergy severity rather than the sole cause of the observed outcomes.

### 4.1. Association Between Maternal Dietary Avoidance and Infant Linear Growth

The study found that infants whose mothers avoided a wide range of foods (≥5 types) had significantly poorer linear growth at 12 months compared to infants whose mothers practiced no avoidance. This association may be explained through several pathways. Maternal nutritional adequacy is crucial for breast milk composition [17,18,19,20], and may be compromised by broad dietary restrictions. However, an equally plausible explanation is that the infant’s underlying severe allergic condition is the primary driver of growth faltering. Significant FA can be associated with chronic gut inflammation, malabsorption, or feeding difficulties, all of which may directly impair growth [21,22,23,24]. Consequently, extensive maternal avoidance likely serves more as a marker of greater initial disease severity rather than the sole cause of growth faltering. The study did not assess breast milk composition and could not fully account for disease activity; thus, we cannot determine the relative contribution of these pathways. Our findings should therefore be interpreted as identifying infants exposed to extensive maternal dietary avoidance as a high-risk group for suboptimal linear growth, warranting close monitoring. It should be noted that the direct comparison between FA low- and high-avoidance subgroups did not reach statistical significance, which may reflect limited statistical power due to the modest sample size within these subgroups. Nevertheless, the effect estimate suggests a trend toward better linear growth in the low-avoidance group, consistent with a graded association across levels of maternal avoidance.

### 4.2. Association Between Maternal Dietary Avoidance and Infant Social–Emotional Risk

The study observed a positive association between the extent of maternal avoidance and infant social–emotional risk scores. Multiple factors may contribute. One possible pathway involves the infant’s own illness experience. Infants with significant FA often suffer from distressing symptoms such as pruritus, gastrointestinal discomfort, and sleep disruption [25,26,27]. These chronic discomforts may affect emotional regulation and social engagement [28,29,30]. Secondly, extensive dietary avoidance can impose considerable parenting stress and anxiety on mothers [31,32,33], which may affect their emotional availability and caregiving sensitivity. This altered maternal state and parent-child interaction could indirectly influence infant social–emotional development [28]. Thus, the findings serve as a clinical alert: infants who undergo extensive maternal dietary avoidance due to FA symptoms constitute a high-risk group not only for nutritional issues but also for social–emotional development. Clinical management should therefore integrate nutritional support, allergic symptom control, and developmental-behavioral surveillance.

### 4.3. Clinical Implications

The results of the present study do not argue against medically necessary allergen avoidance. Rather, they highlight the need for a balanced and comprehensive management strategy for breastfed infants with FA. Infants whose mothers practice extensive dietary avoidance should be recognized as a high-risk group requiring integrated nutritional and developmental support. Several evidence-based strategies can be implemented in clinical practice. First, mothers who eliminate five or more food categories should receive structured nutritional assessment and counseling. Referral to a registered dietitian is recommended to evaluate dietary adequacy and to ensure sufficient intake of calcium, vitamin D, iron, and omega 3 fatty acids. When dietary intake is insufficient, targeted supplementation or the use of hypoallergenic maternal milk substitutes may be considered [21]. Second, infants in this high-risk group warrant enhanced growth monitoring. Monthly anthropometric measurements plotted on WHO growth charts can facilitate early detection of growth faltering, particularly for linear growth, which may be more sensitive to nutritional compromise. Any downward deflection in the length-for-age Z-score should prompt a comprehensive review of both maternal diet and infant feeding practices. Third, routine developmental surveillance using validated screening tools such as the ASQ:SE-2 should be integrated into the follow-up care of these infants. Elevated scores should trigger referral to developmental pediatrics or early intervention services. Given that maternal dietary avoidance may also reflect heightened parenting stress, screening for maternal anxiety and providing appropriate mental health resources or peer support groups may further benefit the mother–infant dyad [33].

In summary, clinical management of food allergy in breastfed infants should extend beyond allergen avoidance alone. A multidisciplinary approach that combines allergy symptom control, maternal nutritional support, systematic infant growth surveillance, and developmental monitoring can help optimize both short-term management and long-term health outcomes.

## 5. Limitations and Future Directions

This study has several limitations. First, as an observational study, confounding by indication is inherent because maternal dietary avoidance was implemented in response to infant symptoms. Maternal avoidance was self-reported, which may introduce recall bias. Second, FA diagnosis relied on clinical assessment without a double-blind, placebo-controlled food challenge, which may introduce some diagnostic misclassification. The demographic questionnaire was adapted from clinical intake forms and has not undergone formal validation; however, the variables collected were largely objective. Third, the achieved sample size (*n* = 75) was substantially lower than the a priori calculated requirement of 145 per group. Consequently, this study should be considered exploratory and underpowered, particularly for subgroup analyses. Non-significant findings should be interpreted cautiously, as the risk of Type II error is elevated. The categorization of maternal avoidance using the sample median (≥5 vs. <5 food categories) is also exploratory, as no established clinical threshold exists. Fourth, follow-up ended at 12 months, preventing assessment of longer-term trajectories. Finally, this was a single-center study conducted in Chongqing, China, and the findings may not be fully generalizable to other populations.

Future research should employ objective diagnostic criteria, enroll larger multicenter cohorts with extended follow-up, and incorporate more comprehensive covariate adjustment. Studies should also examine maternal dietary avoidance as a continuous variable to better characterize potential graded associations.

## 6. Conclusions

Our findings suggest a graded association across levels of maternal dietary avoidance: infants whose mothers avoided five or more food categories showed lower linear growth and higher social–emotional risk scores at 12 months. Given the observational design, these findings should not be interpreted as causal. Rather, extensive maternal avoidance should be recognized as a marker for infants requiring integrated nutritional and developmental surveillance, alongside appropriate allergy management.

## Figures and Tables

**Table 1:** Basic information of the infant at enrollment.

Basic Information	FA Group (*n* = 40)	NFA Group (*n* = 35)	T-Value/χ^2^	*p*-Value
Continuous variable ($\bar{x}$ ± SD)				
Age of enrollment (months)	5.43 ± 1.11	5.02 ± 0.91	1.75	0.09
Birth weight (kg)	3.30 ± 0.37	3.30 ± 0.37	0.05	0.96
Birth length (cm)	49.90 ± 1.27	49.90 ± 1.45	−0.57	0.95
Gestational age (days)	272.96 ± 15.80	273.10 ± 17.63	−0.43	0.97
gravidity	1.55 ± 0.85	1.34 ± 0.87	1.04	0.30
parity	1.18 ± 0.38	1.11 ± 0.32	0.73	0.47
Parental Height Characteristics (cm)				
Maternal height	158.40 ± 4.70	159.50 ± 4.3	−0.97	0.337
Paternal height	171.40 ± 4.60	172.90 ± 5.6	−1.31	0.194
Mid-parental height	164.90 ± 3.60	166.20 ± 3.9	−1.49	0.141
Categorical variables, n (%)				
gender			0.34	0.56
male	21 (52.5)	16 (45.7)		
female	19 (47.5)	19 (54.3)		
Mode of delivery			2.67	0.26
natural delivery	19 (47.5)	23 (65.7)		
caesarean section	20 (50.0)	11 (31.4)		
Complementary feeding			0.07	0.78
Yes	7 (17.5)	7 (20.0)		
No	33 (82.5)	28 (80.0)		

**Table 2:** Characteristics of maternal dietary avoidance in the food allergy group (*n* = 40).

A. Foods Avoided	*n* (%)	B. Number of Food Categories Avoided	*n* (%)
Cow‘s milk/Dairy	34 (85.0)	1	5 (12.5)
Shellfish (shrimp, crab)	32 (80.0)	2	5 (12.5)
Hen’s egg	25 (62.5)	3	7 (17.5)
Tree nuts	24 (60.0)	4	3 (7.5)
Soy	22 (55.0)	5	1 (2.5)
Fish	20 (50.0)	6	7 (17.5)
Peanut	18 (45.0)	7	5 (12.5)
Wheat	11 (27.5)	8	7 (17.5)

**Table 3:** Linear Regression Analysis of Factors Associated with the Number of Food Categories Avoided by Mothers in the FA Group.

Factors	β (95% CI)	*p*-Value
Number of infant allergic symptom systems	0.64 (0.08, 1.21)	0.03
Maternal age (years)	−0.01 (−0.30, 0.28)	0.95
Maternal education level	−0.87 (−2.22, 0.49)	0.20
Gravidity	0.41 (−1.11, 1.92)	0.59
Parity	0.44 (−2.76, 3.64)	0.78
Infant birth weight Z-score	2.42 (−4.39, 9.22)	0.47
Infant birth length Z-score	−1.18 (−5.63, 3.26)	0.59
Infant birth head circumference Z-score	0.57 (−0.62, 1.76)	0.34
Infant birth weight-for-length Z-score	−2.08 (−7.42, 3.27)	0.43

**Table 4:** Longitudinal associations of maternal dietary avoidance extent with infant growth and social–emotional development (Generalized Estimating Equation models).

Comparison Group (Reference: FA High-Avoidance)	Outcome Measure	β (95% CI)	*p*-Value
NFA group	Length-for-age Z-score	0.84 (0.38 to 1.30)	<0.001
FA Low-avoidance group	Length-for-age Z-score	0.50 (−0.09 to 1.08)	0.10
NFA group	ASQ:SE-2 Total Score	−13.34 (−18.39 to −8.30)	<0.001
FA Low-avoidance group	ASQ:SE-2 Total Score	−7.66 (−14.07 to −1.25)	0.02

Note: Models were adjusted for infant age, infant sex, birth weight Z-score, and mid-parental height.

## Data Availability

The data presented in this study are available on request from the corresponding author. The data are not publicly available due to privacy and ethical restrictions.

## Supplementary Materials

The following supporting information can be downloaded at https://www.mdpi.com/article/10.3390/children13050603/s1, Figure S1: Flow diagram of study participants; Table S1: Longitudinal anthropometric Z-scores by maternal avoidance group and infant age; Table S2: Distribution of social–emotional scores by maternal avoidance group; Method S1: Detailed description of data collection procedures and instruments; Method S2: Detailed sample size calculation.

## References

[1] Sampath V., Abrams E.M., Adlou B., Akdis C., Akdis M., Brough H.A., Chan S., Chatchatee P., Chinthrajah R.S., Cocco R.R. (2021). Food allergy across the globe. J. Allergy Clin. Immunol..

[2] Santos A.F., Riggioni C., Agache I., Akdis C.A., Akdis M., Alvarez-Perea A., Alvaro-Lozano M., Ballmer-Weber B., Barni S., Beyer K. (2025). EAACI guidelines on the management of IgE-mediated food allergy. Allergy.

[3] Gamirova A., Berbenyuk A., Levina D., Peshko D., Simpson M.R., Azad M.B., Järvinen K.M., Brough H.A., Genuneit J., Greenhawt M. (2022). Food Proteins in Human Breast Milk and Probability of IgE-Mediated Allergic Reaction in Children During Breastfeeding: A Systematic Review. J. Allergy Clin. Immunol. Pract..

[4] Meyer R., Chebar Lozinsky A., Fleischer D.M., Vieira M.C., Du Toit G., Vandenplas Y., Dupont C., Knibb R., Uysal P., Cavkaytar O. (2020). Diagnosis and management of Non-IgE gastrointestinal allergies in breastfed infants—An EAACI Position Paper. Allergy.

[5] Vandenplas Y., Broekaert I., Domellöf M., Indrio F., Lapillonne A., Pienar C., Ribes-Koninckx C., Shamir R., Szajewska H., Thapar N. (2024). An ESPGHAN Position Paper on the Diagnosis, Management, and Prevention of Cow’s Milk Allergy. J. Pediatr. Gastroenterol. Nutr..

[6] Zhou W., Zhao J., Che H.L., Hong J.G., Hong L., Li H., Li Z.L., Meng J., Sha L., Shao J. (2022). Evidence-based guidelines for food allergy of children in China. Chin. J. Appl. Clin. Pediatr..

[7] Gelsomino M., Liotti L., Barni S., Mori F., Giovannini M., Mastrorilli C., Pecoraro L., Saretta F., Castagnoli R., Arasi S. (2024). Elimination Diets in Lactating Mothers of Infants with Food Allergy. Nutrients.

[8] Bravi F., Di Maso M., Eussen S.R.B.M., Agostoni C., Salvatori G., Profeti C., Tonetto P., Quitadamo P.A., Kazmierska I., Vacca E. (2021). Dietary Patterns of Breastfeeding Mothers and Human Milk Composition: Data from the Italian MEDIDIET Study. Nutrients.

[9] Ding Y., Yang Y., Xu F., Ye M., Hu P., Jiang W., Li F., Fu Y., Xie Z., Zhu Y. (2021). Association between dietary fatty acid patterns based on principal component analysis and fatty acid compositions of serum and breast milk in lactating mothers in Nanjing, China. Food Funct..

[10] Karcz K., Królak-Olejnik B. (2021). Vegan or vegetarian diet and breast milk composition—A systematic review. Crit. Rev. Food Sci. Nutr..

[11] Rio-Aige K., Selma-Royo M., Cabrera-Rubio R., González S., Martínez-Costa C., Castell M., Rodríguez-Lagunas M.J., Collado M.C., Pérez-Cano F.J. (2025). Maternal diet shapes infant microbiota and defensive capacity against infections in early life via differential human milk composition. eBioMedicine.

[12] Sindi A.S., Stinson L.F., Lean S.S., Chooi Y.-H., Leghi G.E., Netting M.J., Wlodek M.E., Muhlhausler B.S., Geddes D.T., Payne M.S. (2022). Effect of a reduced fat and sugar maternal dietary intervention during lactation on the infant gut microbiome. Front. Microbiol..

[13] Ramiro-Cortijo D., Singh P., Herranz Carrillo G., Gila-Díaz A., Martín-Cabrejas M.A., Martin C.R., Arribas S.M. (2023). Association of maternal body composition and diet on breast milk hormones and neonatal growth during the first month of lactation. Front. Endocrinol..

[14] The Group of Standardized Construction for Allergic Diseases, Futang Research Center of Pediatric Development, Allergen Specific Diagnostics Group, Chinese Society of Allergology, Chinese Medical Association, Specialty Society of Allergic Disease Prevention and Control, Chinese Preventive Medicine Association, Committee on Allergy, Beijing Research Association for Chronic Diseases Control and Health Education, Committee on Allergy, Chinese Association of Rehabilitation Medicine (2024). Expert consensus on the diagnosis and management of IgE mediated food allergy in children. Zhonghua Yu Fang Yi Xue Za Zhi.

[15] Bian X., Xie H., Squires J., Chen C.-Y. (2017). ADAPTING A PARENT-COMPLETED, SOCIOEMOTIONAL QUESTIONNAIRE IN CHINA: THE AGES & STAGES QUESTIONNAIRES: SOCIAL-EMOTIONAL. Infant. Ment. Health J..

[16] Vale S.L., Netting M.J., Hornung C.J., Smith J., Roche I., McWilliam V., Hollinshead K., South C., Young A., Rueter K. (2026). ASCIA Guideline: Infant Feeding for Food Allergy Prevention. Clin. Exp. Allergy.

[17] Samuel T.M., Zhou Q., Giuffrida F., Munblit D., Verhasselt V., Thakkar S.K. (2020). Nutritional and Non-nutritional Composition of Human Milk Is Modulated by Maternal, Infant, and Methodological Factors. Front. Nutr..

[18] Binder C., Baumgartner-Parzer S., Gard L.-I., Berger A., Thajer A. (2023). Maternal Diet Influences Human Milk Protein Concentration and Adipose Tissue Marker. Nutrients.

[19] Favara G., Maugeri A., Barchitta M., Lanza E., Magnano San Lio R., Agodi A. (2024). Maternal Lifestyle Factors Affecting Breast Milk Composition and Infant Health: A Systematic Review. Nutrients.

[20] Kankaew S., Briere C.-E. (2025). Maternal Nutrition and Human Milk Nutrients: A Scoping Review. MCN Am. J. Matern. Child. Nurs..

[21] Venter C., Meyer R., Bauer M., Bird J.A., Fleischer D.M., Nowak-Wegrzyn A., Anagnostou A., Vickery B.P., Wang J., Groetch M. (2024). Identifying Children at Risk of Growth and Nutrient Deficiencies in the Food Allergy Clinic. J. Allergy Clin. Immunol. Pract..

[22] Rosow R., Virkud Y.V., Martin V.M., Young M., Su K., Phadke N., Shreffler W.G., Yuan Q. (2023). Longitudinal Assessment of Early Growth in Children with IgE- and Non-IgE-Mediated Food Allergy in a Healthy Infant Cohort. Ann. Allergy Asthma Immunol..

[23] Labrosse R., Graham F., Caubet J.-C. (2020). Non-IgE-Mediated Gastrointestinal Food Allergies in Children: An Update. Nutrients.

[24] Robert E., Al-Hashmi H.A., Al-Mehaidib A., Alsarraf K., Al-Turaiki M., Aldekhail W., Al-Herz W., Alkhabaz A., Bawakid K.O., Elghoudi A. (2024). Symptoms and management of cow’s milk allergy: Perception and evidence. Front. Allergy.

[25] Groetch M., Venter C., Meyer R. (2025). Clinical Presentation and Nutrition Management of Non-IgE-Mediated Food Allergy in Children. Clin. Exp. Allergy.

[26] Anvari S., Miller J., Yeh C.-Y., Davis C.M. (2019). IgE-Mediated Food Allergy. Clin. Rev. Allergy Immunol..

[27] Nemet S., Elbirt D., Mahlab-Guri K., Bezalel-Rosenberg S., Asher I., Talmon A., Rubin L., Ribak Y., Sergienko R., Tal Y. (2023). Food-induced anaphylaxis during infancy is associated with later sleeping and eating disorders. Pediatr. Allergy Immunol..

[28] Cummings A.J., Knibb R.C., King R.M., Lucas J.S. (2010). The psychosocial impact of food allergy and food hypersensitivity in children, adolescents and their families: A review. Allergy.

[29] Fishbein A.B., Mueller K., Kruse L., Boor P., Sheldon S., Zee P., Paller A.S. (2018). Sleep disturbance in children with moderate/severe atopic dermatitis: A case-control study. J. Am. Acad. Dermatol..

[30] Khosravi A., Glińska J., Barańska-Rybak W. (2024). Sleep Efficiency and Neurocognitive Decline in Atopic Dermatitis: A Systematic Review. Acta Derm.-Venereol..

[31] Beken B., Celik V., Gokmirza Ozdemir P., Sut N., Gorker I., Yazicioglu M. (2019). Maternal anxiety and internet-based food elimination in suspected food allergy. Pediatr. Allergy Immunol..

[32] Jung M., Kang U., Kim S., Yoo H.W., Kim H.-Y., Kim M., Lee J.Y., Kim K., Lee E., Kang B.-C. (2023). Psychological Distress and Perceived Burden in Parents of Korean Children with IgE-Mediated Food Allergy. J. Korean Med. Sci..

[33] Yilmaz O., Kacar A.S., Gogebakan E., Can C., Necef I., Mutluer T., Uslu Kizilkan N., Taskiran A.S., Sackesen C. (2022). The relationship between dietary elimination and maternal psychopathology in breastfeeding mothers of infants with food allergy. Pediatr. Allergy Immunol..

