# Supplementary material for: The Impact of Maternal Dietary Avoidance During Breastfeeding on Physical Growth and Social–Emotional Development in Infants with Food Allergies: A Prospective Cohort Study
Source: Children (Basel). 2026 Apr 27;13(5):603. doi: 10.3390/children13050603 (PMC13204089; doi:10.3390/children13050603)
Supplement: Supplementary file 1 [file children-13-00603-s001.zip › children-4246594-supplementary.pdf]

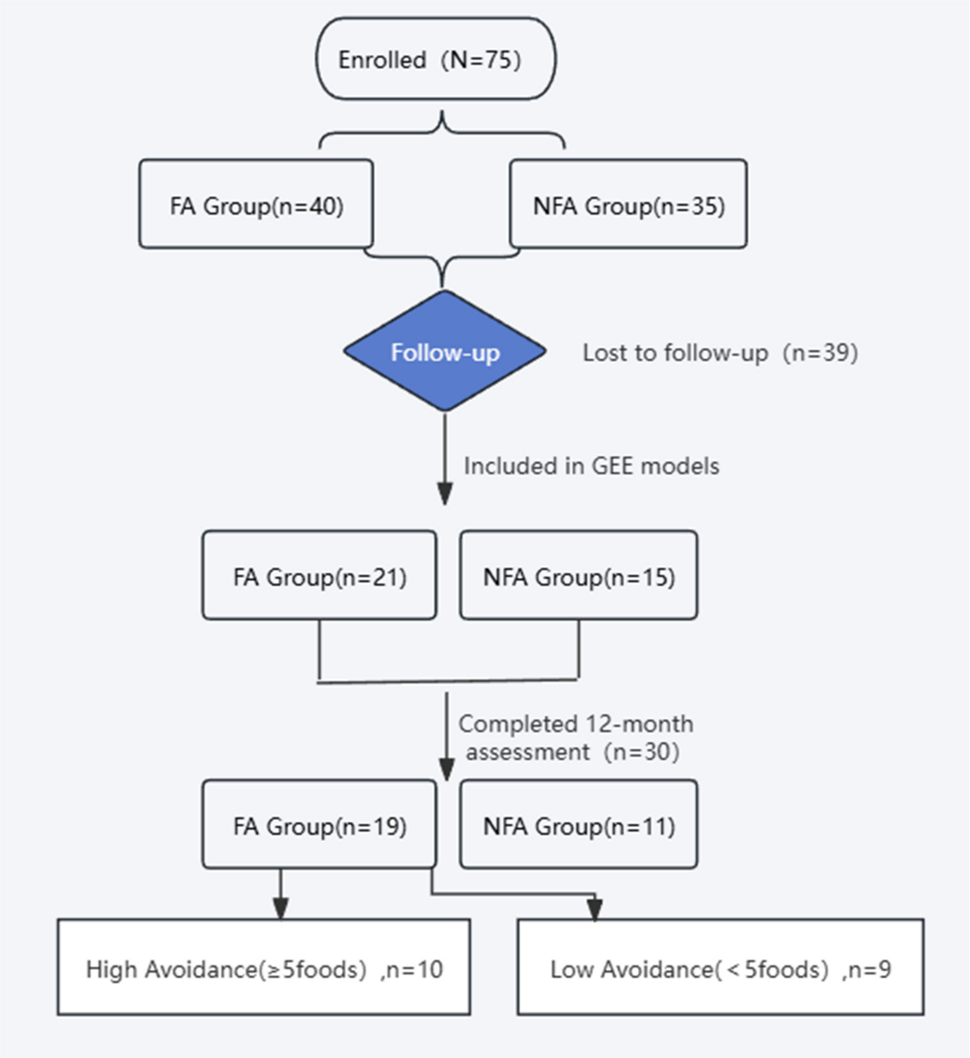

Supplementary Figure S1. Flow diagram of study participants. FA, food allergy; NFA, non-food allergy. The diagram shows the flow of participants from enrollment and group allocation, through follow-up visits at 2, 4, 6, and 8 months after enrollment (corresponding to infant ages of approximately 6, 8, 10, and 12 months), including attrition, to final inclusion in the longitudinal analyses (Generalized Estimating Equations, GEE). Sample sizes for the FA high- and low-avoidance subgroups (n=10 and n=9, respectively) indicate infants who completed the 12-month assessment.

Supplementary Table S1. Longitudinal anthropometric Z-scores by maternal avoidance group and infant age.

Group	Age (months)	n	Weight Z-score (Mean±SD)	Length Z-score (Mean±SD)	Head Circumference Z-score (Mean±SD)	Weight-for-Length Z-score (Mean±SD)
FA High-avoidance (avoid ≥5 types)	6	6	0.35 ± 0.47	-0.26 ± 0.24	0.38 ± 0.73	0.73 ± 0.56
	8	10	-0.18 ± 1.17	-1.04 ± 0.88	-0.10 ± 0.91	0.59 ± 0.93
	10	11	-0.49 ± 1.10	-1.07 ± 0.90	-0.50 ± 0.86	0.10 ± 1.04
	12	10	-0.40 ± 0.97	-1.25 ± 0.71	-0.40 ± 0.65	0.27 ± 1.03
FA Low-avoidance (avoid <5 types)	6	7	-0.25 ± 0.79	-0.62 ± 0.92	-0.27 ± 0.98	0.25 ± 0.91
	8	12	-0.29 ± 0.87	-0.45 ± 0.86	-0.29 ± 1.27	0.17 ± 0.88
	10	11	-0.26 ± 0.83	-0.47 ± 1.12	-0.25 ± 1.33	0.02 ± 0.96
	12	9	-0.07 ± 0.79	-0.44 ± 0.81	-0.01 ± 1.23	0.18 ± 0.85
NFA Group (No avoidance)	6	14	0.53 ± 0.63	0.23 ± 0.44	-0.04 ± 0.75	0.60 ± 0.76
	8	23	0.04 ± 0.87	-0.22 ± 1.04	-0.16 ± 0.94	0.29 ± 0.87

Group	Age (months)	n	Weight Z-score (Mean±SD)	Length Z-score (Mean±SD)	Head Circumference Z-score (Mean±SD)	Weight-for-Length Z-score (Mean±SD)
	10	14	-0.10 ± 0.36	-0.07 ± 0.79	-0.25 ± 0.33	-0.07 ± 0.82
	12	11	0.17 ± 0.61	0.25 ± 0.90	0.11 ± 1.02	0.08 ± 0.62

Abbreviations: FA, food allergy; NFA, non-food allergy; SD, standard deviation.

Supplementary Table S2. Distribution of Social-Emotional Scores by Maternal Avoidance Group

Group	Age (months)	n	ASQ:SE-2 Score, Median (IQR)
FA High-avoidance (≥5 types)	8	10	30.0
	10	10	22.0
	12	10	30.0
FA Low-avoidance (<5 types)	8	9	20.0
	10	10	20.0
	12	9	15.0
NFA group	8	23	15.0
	10	12	10.0
	12	11	10.0

Abbreviations: FA, food allergy; NFA, non-food allergy; ASQ:SE-2, Ages & Stages Questionnaires: Social-Emotional, Second Edition.

Supplementary Method S1: Detailed Description of Data Collection Procedures and Instruments

1. Anthropometric Measurement Protocol

All anthropometric measurements were performed by two experienced nurses from the Child Health Care Department of Children's Hospital of Chongqing Medical University, Chongqing, China following standardized procedures to ensure accuracy

and reproducibility.

Weight: Measured using a calibrated lever scale with an accuracy of 0.05 kg. Infants were weighed naked or wearing only a dry diaper.

Length: Measured in the supine position using a standard infant measuring bed with a fixed headboard and a movable footboard, accurate to 0.1 cm. The infant's head was positioned against the headboard with the eyes looking vertically upward, knees held down gently, and feet positioned flat against the footboard.

Head Circumference: Measured using a non-stretchable, flexible tape measure accurate to 0.1 cm. The tape was placed around the head at the maximum occipitofrontal circumference, above the eyebrows and ears, and over the most prominent part of the occiput.

All measurements were taken in duplicate. If the two readings differed by more than a predefined tolerance (0.1 kg for weight, 0.5 cm for length and head circumference), a third measurement was taken. The average of the two closest readings was used for analysis. Z-scores (weight-for-age, length-for-age, head circumference-for-age, weight-for-length) were calculated using the WHO Anthro software (version 3.2.2, 2011) based on the WHO Child Growth Standards.

2. Assessment of Infant Social-Emotional Development: ASQ:SE-2

2.1 Instrument Description

The Ages & Stages Questionnaires: Social-Emotional, Second Edition (ASQ:SE-2) is a parent-completed screening tool designed to identify infants and young children at risk for social-emotional delays. The Chinese version used in this study has undergone localization, and its reliability and validity have been established for use in China [Bian et al., 2017, cited in main text as Ref 13].

2.2 Domains and Items

The questionnaire assesses seven key behavioral domains:

A. Self-Regulation: The child's ability to calm or settle.

B. Compliance: The child's ability to follow rules and routines.

C. Adaptive Functioning: The child's ability to adapt to changes and daily activities (e.g., sleeping, eating).

D. Autonomy: The child's ability to act independently.

E. Affect: The child's expression and range of emotions.

F. Social-Communication: The child's use of gestures, sounds, and words to interact.

G. Interaction with People: The child's ability to engage with caregivers and others.

2.3 Forms and Administration

Age-appropriate forms were used at each follow-up visit:

For infants aged 3–8 months, the 6-month interval form (23 items) was administered.

For infants aged 9–12 months, the 12-month interval form (27 items) was

administered.

Parents were asked to observe their child's behavior over the preceding 1-2 weeks and mark the response that best described the frequency of each behavior: "Never or Rarely," "Sometimes," "Often," or "Always or Most of the Time."

2.4 Scoring System

Each item is assigned a score of 0, 5, 10, or 15 points, corresponding to the response categories from lowest to highest concern.

The scores for all items are summed to yield a Total Score.

Interpretation of Total Scores (Cut-offs):

A. For the 6-month form:

Below Cutoff (Typical Development): Total Score < 30

Close to Cutoff (Monitor): Total Score 30 – 44

Above Cutoff (Further Evaluation Suggested): Total Score ⩾ 45

B. For the 12-month form:

Below Cutoff (Typical Development): Total Score < 40

Close to Cutoff (Monitor): Total Score 40 – 49

Above Cutoff (Further Evaluation Suggested): Total Score ⩾ 50

A higher ASQ:SE-2 Total Score indicates greater concern regarding the child's social-emotional development and a higher likelihood of delay.

3. Maternal Dietary Avoidance Assessment

A structured questionnaire was used to assess maternal dietary avoidance. Mothers were presented with a list of eight common allergenic food categories: Cow's milk and dairy products, Hen's egg, Peanut, Tree nuts (e.g., almonds, walnuts), Wheat,Soy, Fish, Crustacean shellfish (shrimp, crab).

For each category, mothers were asked: "Have you completely avoided consuming this type of food for a period of 2 weeks or more since your infant's allergy symptoms began?" Avoidance was strictly defined as complete elimination for ⩾2 weeks prior to the assessment. The total number of avoided categories was summed for each mother.

4. Food Allergy Symptom Assessment

The symptom questionnaire was adapted from the Consensus of Chinese Experts on the Diagnosis and Treatment of Allergic Diseases in Infants [Zhou et al., 2022, cited in main text as Ref 12]. It catalogued symptoms across four systems:

A. Skin: Eczema, urticaria (hives), angioedema, itching.

B. Gastrointestinal: Vomiting, diarrhea, bloody stools, constipation, abdominal pain, food refusal.

C. Respiratory: Rhinorrhea, sneezing, nasal congestion, cough, wheezing, difficulty breathing.

D. Behavioral: Fussiness, crying, sleep disturbance.

Parents indicated which symptoms their infant experienced and their temporal relationship to food ingestion. The number of symptomatic systems (ranging from 1 to 4) was recorded as an indicator of symptom diversity.

Supplementary Method S2. Detailed Sample Size Calculation

Sample size was calculated using PASS software (version 2021, NCSS, LLC, Kaysville, UT, USA) based on the primary outcome of length-for-age Z-score at 12 months.

Input parameters:

Expected mean difference between FA and NFA groups: -0.5

Standard deviation in FA group: 1.5

Standard deviation in NFA group: 1.4

Significance level (α): 0.05 (two-sided)

Power ($1-\beta$): 80%

These estimates were derived from our preliminary research findings (30).

Calculation result:

Required sample size per group: 123 mother-infant pairs

Adjustment for loss to follow-up:

Anticipated dropout rate: 15%

Final target sample size per group: 123 / (1 - 0.15) = 145 mother-infant pairs

Total target sample size: 290 mother-infant pairs

Actual enrollment:

FA group: 40 participants (27.6% of target)

NFA group: 35 participants (24.1% of target)

Total: 75 participants (25.9% of target)

This substantial shortfall in sample size limits statistical power, particularly for subgroup analyses within the FA group (high-avoidance vs. low-avoidance). Therefore, this study should be considered exploratory, and findings require confirmation in larger multi-center cohorts.
